# Supplementary material for: Digging for DNA at depth: rapid universal metabarcoding surveys (RUMS) as a tool to detect coral reef biodiversity across a depth gradient
Source: PeerJ. 2019 Feb 6;7:e6379. doi: 10.7717/peerj.6379 (PMC6368839; doi:10.7717/peerj.6379)
Supplement: Supplemental Information 1 [file peerj-07-6379-s001.docx]

Taxonomy-dependent analyses based on 18S

**'DepthxYear' for pairs of levels of factor 'Depth'**

*Resemblance worksheet*

Name: Jacc Resem Matrix

Data type: Similarity

Selection: All

Transform: Presence/absence

Resemblance: S7 Jaccard

Sums of squares type: Type III (partial)

Fixed effects sum to zero for mixed terms

Permutation method: Permutation of residuals under a reduced model

Number of permutations: 9999

*Factors*

Name Type Levels

Reef Fixed 2

Depth Fixed 4

Year Fixed 2

*PAIR-WISE TESTS*

Term 'DepthxYear' for pairs of levels of factor 'Depth'

Within level '2016' of factor 'Year'

Unique

Groups t P(perm) perms P(MC)

10, 20 1.2209 0.0524 9840 0.1714

10, 30 1.2632 0.0157 9871 0.1106

10, 40 1.0626 0.2988 6692 0.3465

20, 30 1.0779 0.254 9866 0.3197

20, 40 0.96602 0.5535 3030 0.4885

30, 40 0.99616 0.5292 7497 0.4382

*Denominators*

Groups Denominator Den.df

10, 20 1*Res 9

10, 30 1*Res 11

10, 40 1*Res 8

20, 30 1*Res 10

20, 40 1*Res 7

30, 40 1*Res 9

*Average Similarity between/within groups*

10 20 30 40

10 21.006

20 13.552 13.762

30 19.345 17.463 23.053

40 17.149 15.14 20.162 15.792

Within level '2017' of factor 'Year'

Unique

Groups t P(perm) perms P(MC)

10, 20 1.0621 0.2252 35 0.3637

10, 30 1.0872 0.1133 35 0.3367

10, 40 1.2361 0.0595 35 0.2

20, 30 1.0497 0.2579 35 0.3912

20, 40 1.3705 0.0557 35 0.1401

30, 40 1.3149 0.025 35 0.1476

*Denominators*

Groups Denominator Den.df

10, 20 1*Res 5

10, 30 1*Res 6

10, 40 1*Res 6

20, 30 1*Res 5

20, 40 1*Res 5

30, 40 1*Res 6

*Average Similarity between/within groups*

10 20 30 40

10 11.539

20 8.3861 8.4334

30 5.7154 4.8411 4.3519

40 17.037 12.641 10.921 34.973

**'DepthxYear' for pairs of levels of factor 'Year'**

*Resemblance worksheet*

Name: Jacc Resem Matrix

Data type: Similarity

Selection: All

Transform: Presence/absence

Resemblance: S7 Jaccard

Sums of squares type: Type III (partial)

Fixed effects sum to zero for mixed terms

Permutation method: Permutation of residuals under a reduced model

Number of permutations: 9999

*Factors*

Name Type Levels

Reef Fixed 2

Depth Fixed 4

Year Fixed 2

*PAIR-WISE TESTS*

Term 'DepthxYear' for pairs of levels of factor 'Year'

Within level '10' of factor 'Depth'

Unique

Groups t P(perm) perms P(MC)

2016, 2017 1.247 0.0141 6666 0.1592

*Denominators*

Groups Denominator Den.df

2016, 2017 1*Res 8

*Average Similarity between/within groups*

2016 2017

2016 21.006

2017 11.42 11.539

Within level '20' of factor 'Depth'

Unique

Groups t P(perm) perms P(MC)

2016, 2017 1.1594 0.1448 1259 0.2546

*Denominators*

Groups Denominator Den.df

2016, 2017 1*Res 6

*Average Similarity between/within groups*

2016 2017

2016 13.762

2017 9.3735 8.4334

Within level '30' of factor 'Depth'

Unique

Groups t P(perm) perms P(MC)

2016, 2017 1.3432 0.0007 7484 0.0867

*Denominators*

Groups Denominator Den.df

2016, 2017 1*Res 9

*Average Similarity between/within groups*

2016 2017

2016 23.053

2017 4.8625 4.3519

Within level '40' of factor 'Depth'

Unique

Groups t P(perm) perms P(MC)

2016, 2017 1.498 0.0293 35 0.0749

*Denominators*

Groups Denominator Den.df

2016, 2017 1*Res 6

*Average Similarity between/within groups*

2016 2017

2016 15.792

2017 13.291 34.973

**'ReefxDepth' for pairs of levels of factor 'Depth'**

*Resemblance worksheet*

Name: Jacc Resem Matrix

Data type: Similarity

Selection: All

Transform: Presence/absence

Resemblance: S7 Jaccard

Sums of squares type: Type III (partial)

Fixed effects sum to zero for mixed terms

Permutation method: Permutation of residuals under a reduced model

Number of permutations: 9999

*Factors*

Name Type Levels

Reef Fixed 2

Depth Fixed 4

Year Fixed 2

*PAIR-WISE TESTS*

Term 'ReefxDepth' for pairs of levels of factor 'Depth'

Within level 'Cape_Hedo' of factor 'Reef'

Unique

Groups t P(perm) perms P(MC)

10, 20 1.0359 0.3957 9708 0.3785

10, 30 1.2097 0.0185 9859 0.1537

10, 40 1.1215 0.1481 9869 0.2632

20, 30 1.0194 0.4608 9772 0.4079

20, 40 1.169 0.1168 9759 0.2283

30, 40 1.1088 0.1398 9859 0.2724

*Denominators*

Groups Denominator Den.df

10, 20 1*Res 8

10, 30 1*Res 11

10, 40 1*Res 11

20, 30 1*Res 9

20, 40 1*Res 9

30, 40 1*Res 12

*Average Similarity between/within groups*

10 20 30 40

10 15.897

20 12.612 9.5481

30 9.7337 9.0655 8.2735

40 16.198 12.583 12.429 18.473

Within level 'Rukan' of factor 'Reef'

Unique

Groups t P(perm) perms P(MC)

10, 20 1.2617 0.0526 35 0.1723

10, 30 1.355 0.0283 35 0.1147

20, 30 1.2394 0.0308 35 0.1892

*Denominators*

Groups Denominator Den.df

10, 20 1*Res 6

10, 30 1*Res 6

20, 30 1*Res 6

*Average Similarity between/within groups*

10 20 30

10 17.819

20 9.2473 13.658

30 18.805 18.802 35.722

**'ReefxDepth' for pairs of levels of factor 'Reef'**

*Resemblance worksheet*

Name: Jacc Resem Matrix

Data type: Similarity

Selection: All

Transform: Presence/absence

Resemblance: S7 Jaccard

Sums of squares type: Type III (partial)

Fixed effects sum to zero for mixed terms

Permutation method: Permutation of residuals under a reduced model

Number of permutations: 9999

*Factors*

Name Type Levels

Reef Fixed 2

Depth Fixed 4

Year Fixed 2

*PAIR-WISE TESTS*

Term 'ReefxDepth' for pairs of levels of factor 'Reef'

Within level '10' of factor 'Depth'

Unique

Groups t P(perm) perms P(MC)

Cape_Hedo, Rukan 1.1243 0.1327 6587 0.2777

*Denominators*

Groups Denominator Den.df

Cape_Hedo, Rukan 1*Res 8

*Average Similarity between/within groups*

Cape_Hedo Rukan

Cape_Hedo 15.897

Rukan 13.906 17.819

Within level '20' of factor 'Depth'

Unique

Groups t P(perm) perms P(MC)

Cape_Hedo, Rukan 1.0812 0.2604 1257 0.3339

*Denominators*

Groups Denominator Den.df

Cape_Hedo, Rukan 1*Res 6

*Average Similarity between/within groups*

Cape_Hedo Rukan

Cape_Hedo 9.5481

Rukan 11.151 13.658

Within level '30' of factor 'Depth'

Unique

Groups t P(perm) perms P(MC)

Cape_Hedo, Rukan 1.2015 0.0283 7565 0.1928

*Denominators*

Groups Denominator Den.df

Cape_Hedo, Rukan 1*Res 9

*Average Similarity between/within groups*

Cape_Hedo Rukan

Cape_Hedo 8.2735

Rukan 11.912 35.722

Taxonomy-independent analyses based on 18S OTUs for combined Demospongia and Anthozoa

**'ReefxDepth' for pairs of levels of factor 'Depth'**

*Resemblance worksheet*

Name: Resem1

Data type: Similarity

Selection: All

Transform: Presence/absence

Resemblance: S7 Jaccard (+d)

Sums of squares type: Type III (partial)

Fixed effects sum to zero for mixed terms

Permutation method: Permutation of residuals under a reduced model

Number of permutations: 9999

*Factors*

Name Type Levels

Reef Fixed 2

Depth Fixed 4

Year Fixed 2

*PAIR-WISE TESTS*

Term 'ReefxDepth' for pairs of levels of factor 'Depth'

Within level 'Cape_Hedo' of factor 'Reef'

Unique

Groups t P(perm) perms P(MC)

10, 20 1.0252 0.4652 9684 0.4037

10, 30 1.2794 0.0307 9837 0.1236

10, 40 1.0044 0.4396 9852 0.4269

20, 30 1.6539 0.0027 8722 0.021

20, 40 1.2645 0.0621 9440 0.1427

30, 40 0.94193 0.6372 9583 0.5005

*Denominators*

Groups Denominator Den.df

10, 20 1*Res 8

10, 30 1*Res 11

10, 40 1*Res 11

20, 30 1*Res 9

20, 40 1*Res 9

30, 40 1*Res 12

*Average Similarity between/within groups*

10 20 30 40

10 9.9863

20 8.8496 9.0567

30 18.464 12.657 39.913

40 15.533 11.045 34.44 24.866

Within level 'Rukan' of factor 'Reef'

Unique

Groups t P(perm) perms P(MC)

10, 20 1.2218 0.1735 18 0.2267

10, 30 1.4125 0.0298 18 0.0995

20, 30 0.74346 1 6 0.676

*Denominators*

Groups Denominator Den.df

10, 20 1*Res 6

10, 30 1*Res 6

20, 30 1*Res 6

*Average Similarity between/within groups*

10 20 30

10 16.026

20 20.159 38.889

30 16.875 47.083 42.222

**'ReefxDepth' for pairs of levels of factor 'Reef'**

*Resemblance worksheet*

Name: Resem1

Data type: Similarity

Selection: All

Transform: Presence/absence

Resemblance: S7 Jaccard (+d)

Sums of squares type: Type III (partial)

Fixed effects sum to zero for mixed terms

Permutation method: Permutation of residuals under a reduced model

Number of permutations: 9999

*Factors*

Name Type Levels

Reef Fixed 2

Depth Fixed 4

Year Fixed 2

*PAIR-WISE TESTS*

Term 'ReefxDepth' for pairs of levels of factor 'Reef'

Within level '10' of factor 'Depth'

Unique

Groups t P(perm) perms P(MC)

Cape_Hedo, Rukan 1.0368 0.3628 6627 0.3871

*Denominators*

Groups Denominator Den.df

Cape_Hedo, Rukan 1*Res 8

*Average Similarity between/within groups*

Cape_Hedo Rukan

Cape_Hedo 9.9863

Rukan 11.662 16.026

Within level '20' of factor 'Depth'

Unique

Groups t P(perm) perms P(MC)

Cape_Hedo, Rukan 1.3093 0.0169 805 0.1501

*Denominators*

Groups Denominator Den.df

Cape_Hedo, Rukan 1*Res 6

*Average Similarity between/within groups*

Cape_Hedo Rukan

Cape_Hedo 9.0567

Rukan 12.858 38.889

Within level '30' of factor 'Depth'

Unique

Groups t P(perm) perms P(MC)

Cape_Hedo, Rukan 1.3886 0.0183 931 0.1092

*Denominators*

Groups Denominator Den.df

Cape_Hedo, Rukan 1*Res 9

*Average Similarity between/within groups*

Cape_Hedo Rukan

Cape_Hedo 39.913

Rukan 45.15 42.222

**'DepthxYear' for pairs of levels of factor 'Depth'**

*Resemblance worksheet*

Name: Resem1

Data type: Similarity

Selection: All

Transform: Presence/absence

Resemblance: S7 Jaccard (+d)

Sums of squares type: Type III (partial)

Fixed effects sum to zero for mixed terms

Permutation method: Permutation of residuals under a reduced model

Number of permutations: 9999

*Factors*

Name Type Levels

Reef Fixed 2

Depth Fixed 4

Year Fixed 2

*PAIR-WISE TESTS*

Term 'DepthxYear' for pairs of levels of factor 'Depth'

Within level '2016' of factor 'Year'

Unique

Groups t P(perm) perms P(MC)

10, 20 1.0143 0.4522 9839 0.4238

10, 30 1.1372 0.1538 9899 0.2448

10, 40 0.95416 0.6848 5268 0.5095

20, 30 1.0198 0.4342 9801 0.408

20, 40 1.1537 0.1228 1299 0.2604

30, 40 1.0042 0.4797 6237 0.4366

*Denominators*

Groups Denominator Den.df

10, 20 1*Res 9

10, 30 1*Res 11

10, 40 1*Res 8

20, 30 1*Res 10

20, 40 1*Res 7

30, 40 1*Res 9

*Average Similarity between/within groups*

10 20 30 40

10 13.051

20 17.004 21.955

30 17.284 27.094 25.91

40 13.517 16.817 18.092 11.813

Within level '2017' of factor 'Year'

Unique

Groups t P(perm) perms P(MC)

10, 20 1.0795 0.1717 35 0.3456

10, 30 1.8958 0.0307 8 0.0288

10, 40 1.2773 0.1108 15 0.1903

20, 30 2.2312 0.0278 8 0.0172

20, 40 1.3908 0.1418 15 0.1462

30, 40 1 1 1 0.3548

*Denominators*

Groups Denominator Den.df

10, 20 1*Res 5

10, 30 1*Res 6

10, 40 1*Res 6

20, 30 1*Res 5

20, 40 1*Res 5

30, 40 1*Res 6

*Average Similarity between/within groups*

10 20 30 40

10 8.3128

20 8.0139 12.749

30 17.5 16.389 100

40 13.797 13.021 75.862 51.724

**'DepthxYear' for pairs of levels of factor 'Year'**

*Resemblance worksheet*

Name: Resem1

Data type: Similarity

Selection: All

Transform: Presence/absence

Resemblance: S7 Jaccard (+d)

Sums of squares type: Type III (partial)

Fixed effects sum to zero for mixed terms

Permutation method: Permutation of residuals under a reduced model

Number of permutations: 9999

*Factors*

Name Type Levels

Reef Fixed 2

Depth Fixed 4

Year Fixed 2

*PAIR-WISE TESTS*

Term 'DepthxYear' for pairs of levels of factor 'Year'

Within level '10' of factor 'Depth'

Unique

Groups t P(perm) perms P(MC)

2016, 2017 0.98998 0.5447 5257 0.4486

*Denominators*

Groups Denominator Den.df

2016, 2017 1*Res 8

*Average Similarity between/within groups*

2016 2017

2016 13.051

2017 11.016 8.3128

Within level '20' of factor 'Depth'

Unique

Groups t P(perm) perms P(MC)

2016, 2017 1.2079 0.0449 805 0.2232

*Denominators*

Groups Denominator Den.df

2016, 2017 1*Res 6

*Average Similarity between/within groups*

2016 2017

2016 21.955

2017 11.86 12.749

Within level '30' of factor 'Depth'

Unique

Groups t P(perm) perms P(MC)

2016, 2017 1.7555 0.0123 1420 0.0262

*Denominators*

Groups Denominator Den.df

2016, 2017 1*Res 9

*Average Similarity between/within groups*

2016 2017

2016 25.91

2017 46.569 100

Within level '40' of factor 'Depth'

Unique

Groups t P(perm) perms P(MC)

2016, 2017 1.1426 0.1367 15 0.2908

*Denominators*

Groups Denominator Den.df

2016, 2017 1*Res 6

*Average Similarity between/within groups*

2016 2017

2016 11.813

2017 19.689 51.724
