# Supplementary figures and images for: Digging for DNA at depth: rapid universal metabarcoding surveys (RUMS) as a tool to detect coral reef biodiversity across a depth gradient
Source: PeerJ. 2019 Feb 6;7:e6379. doi: 10.7717/peerj.6379 (PMC6368839; doi:10.7717/peerj.6379)

**a**

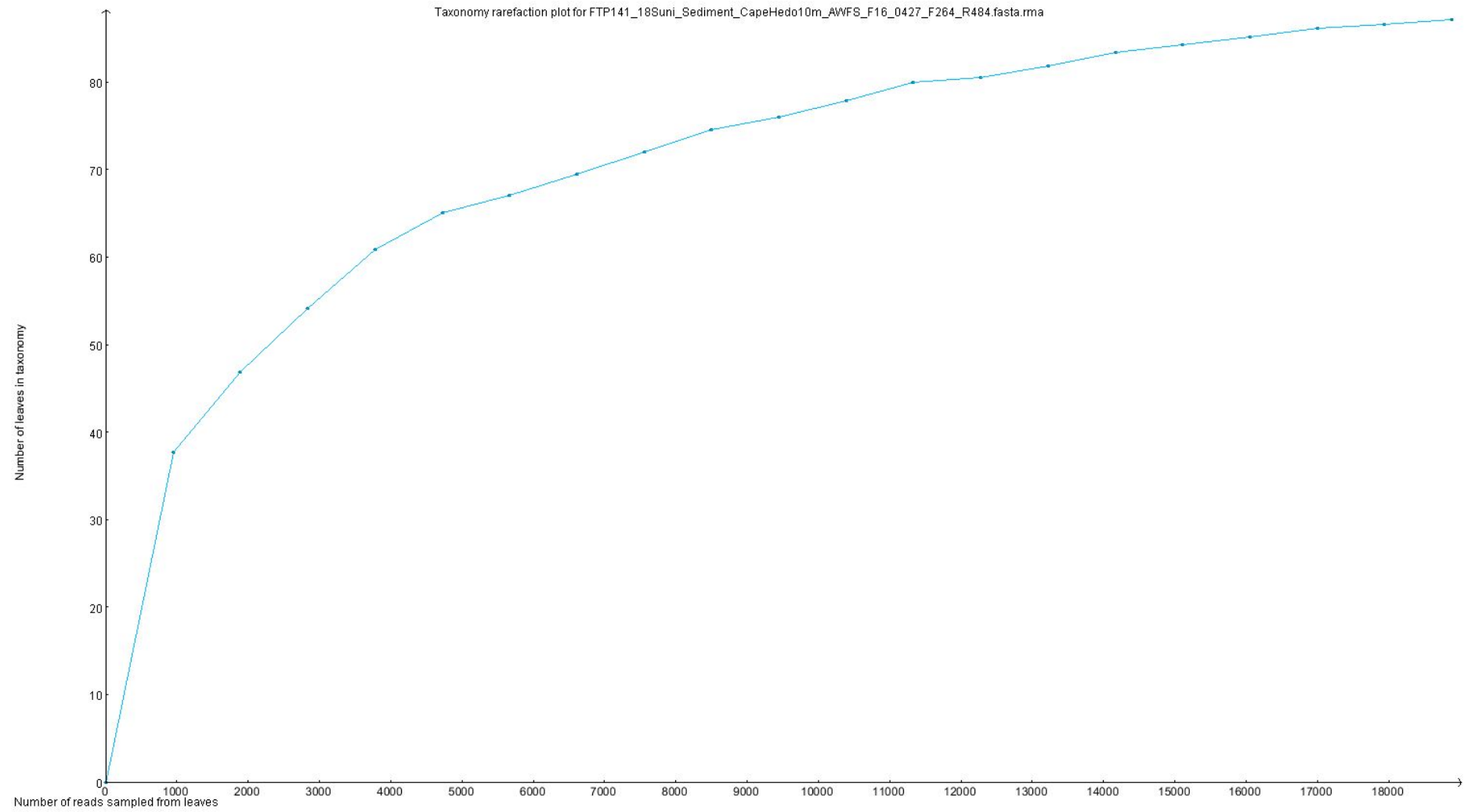

**b**

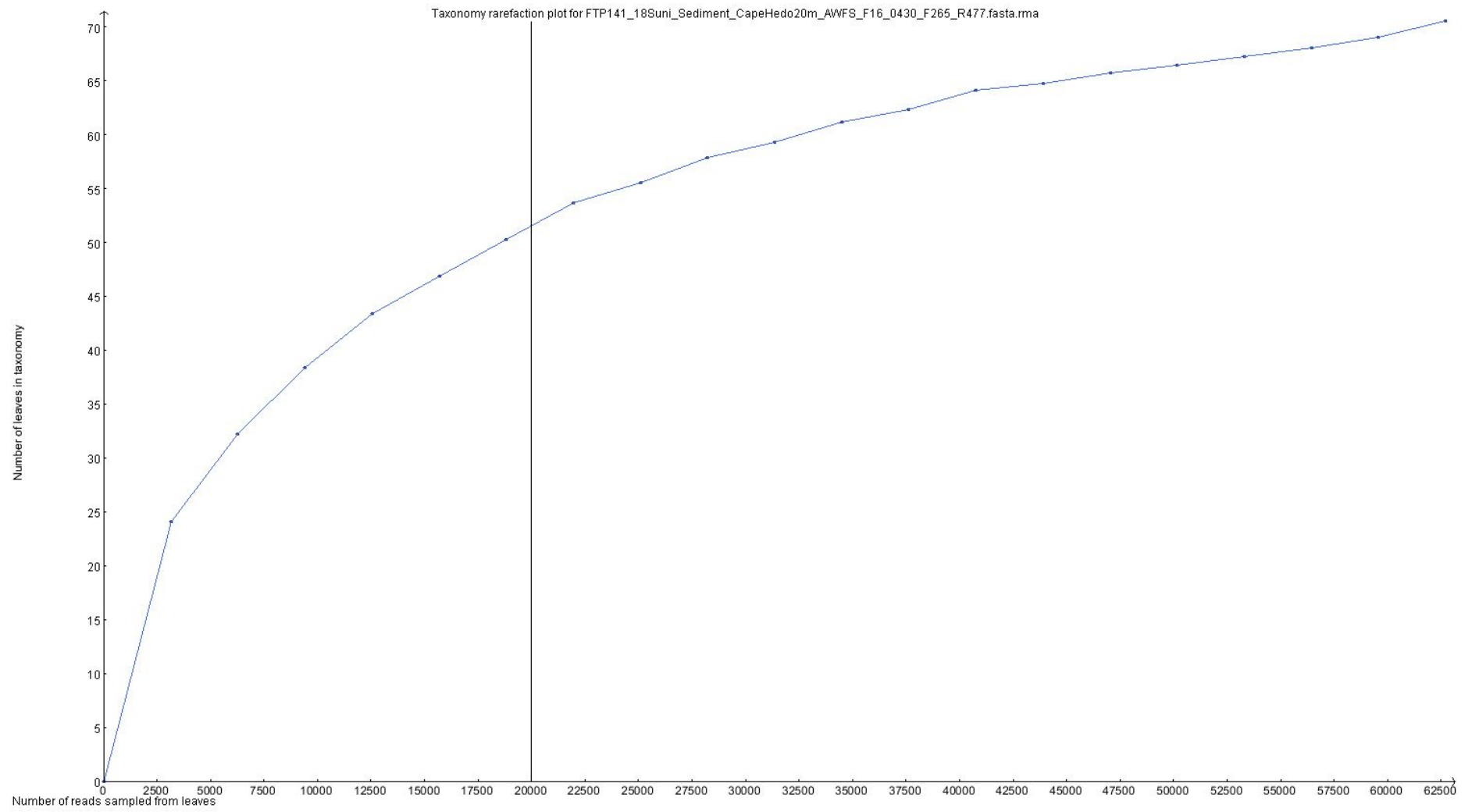

**c**

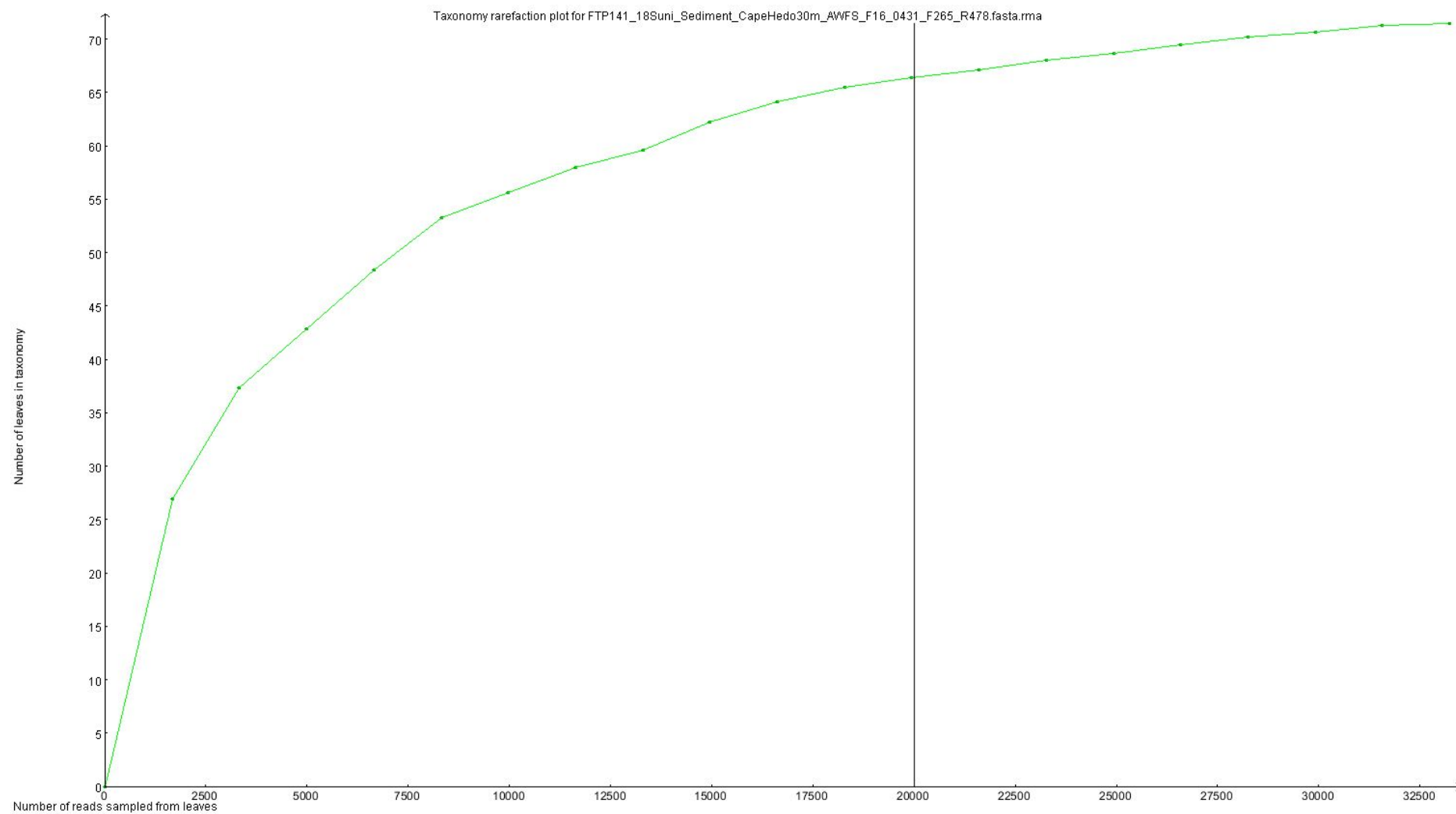

**d**

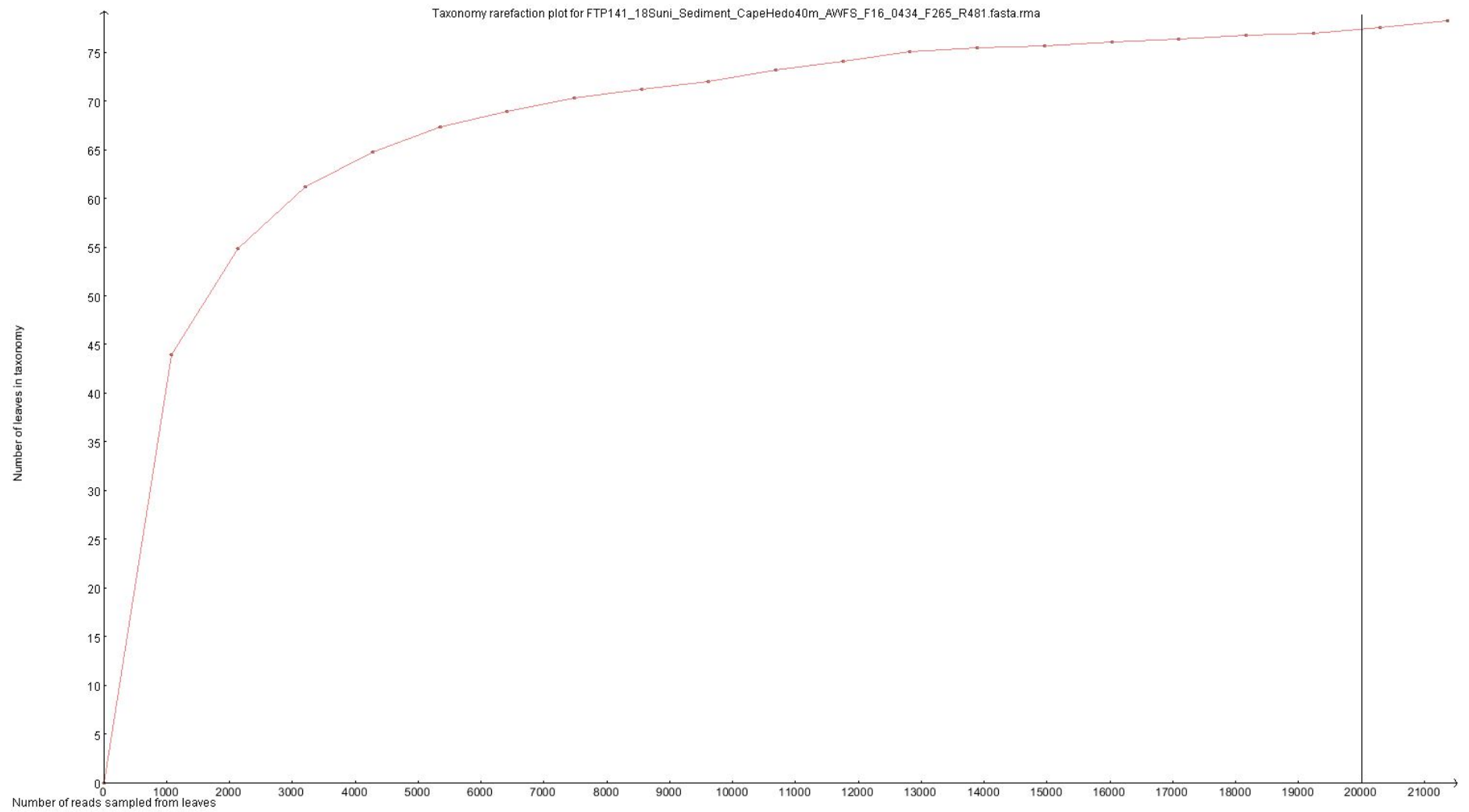

e

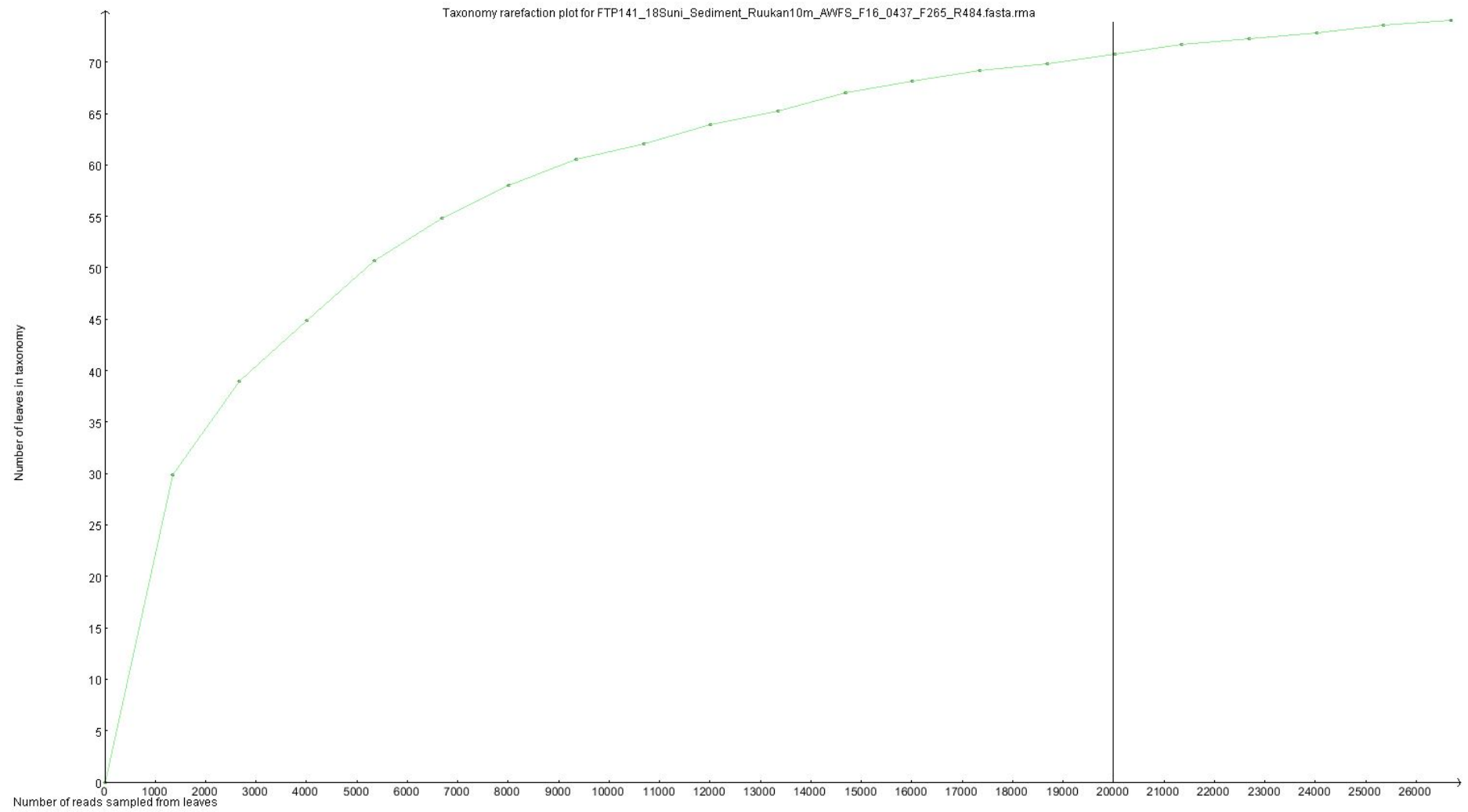

f

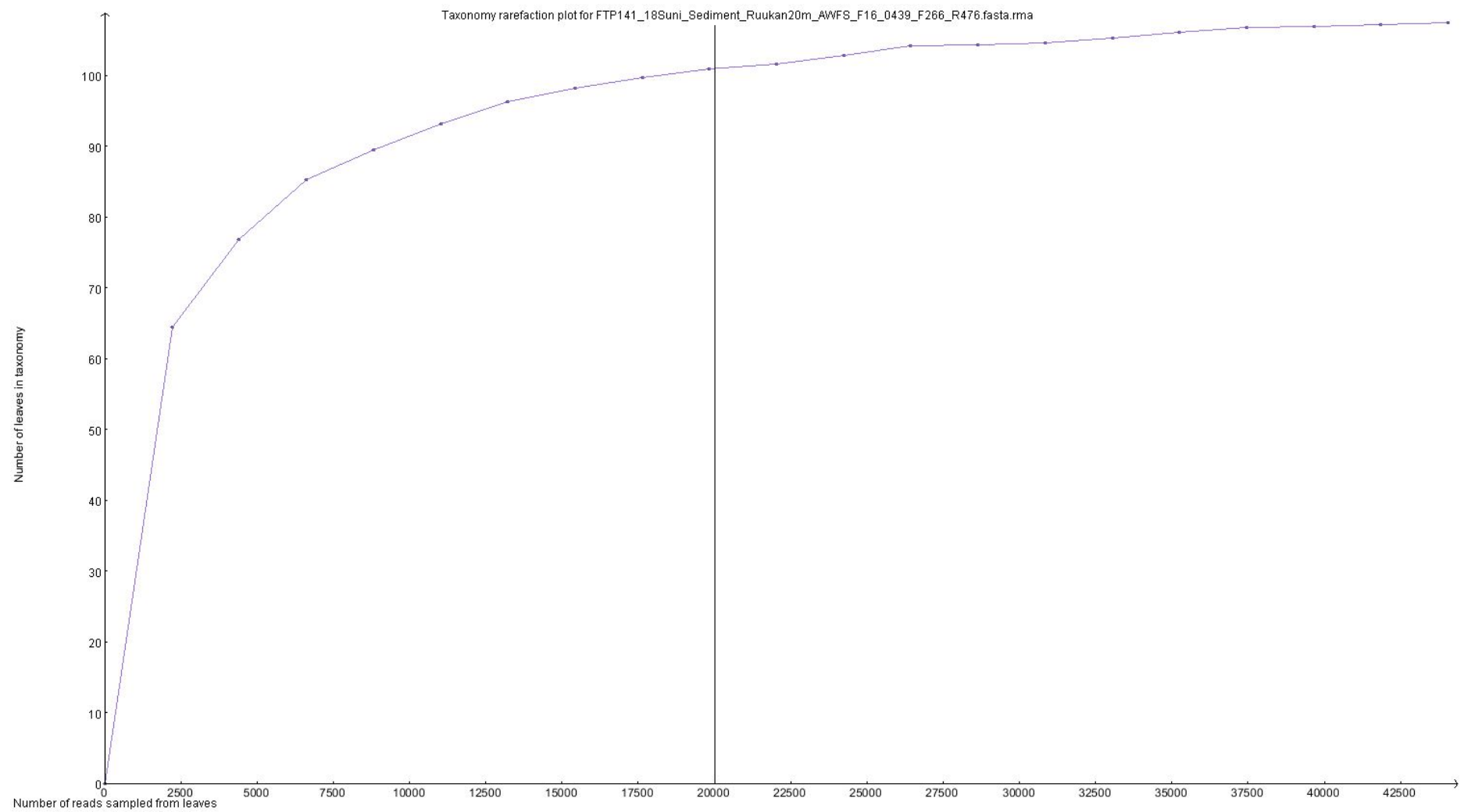

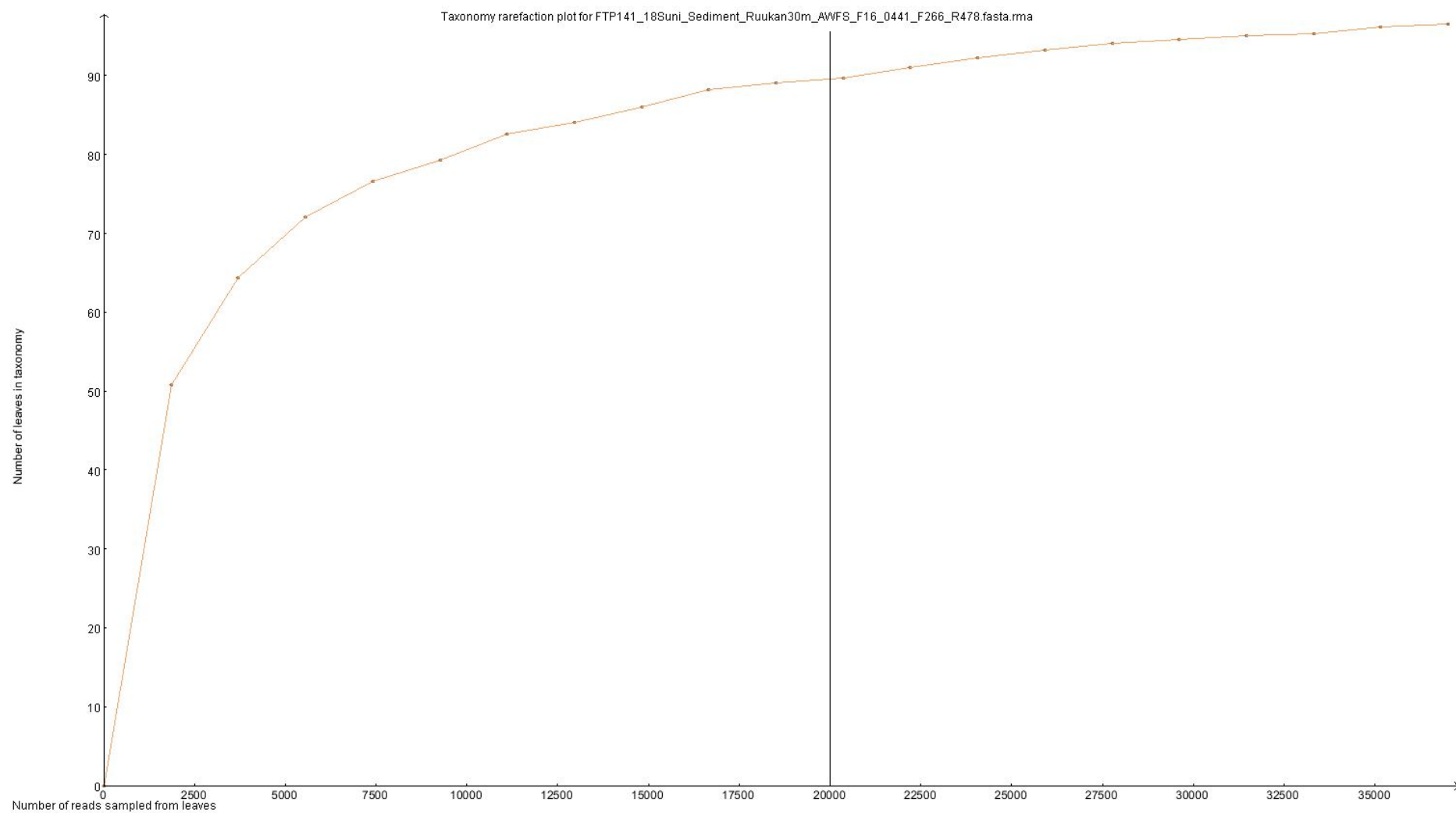

Supplement: Supplemental Information 2 — Rarefaction assignment curves at the family-level based on unfiltered 18S rRNA sequences (a to g) for select sediment samples collected from two coral reefs (10 m, 20 m, 30 m, 40 m) in Okinawa, Japan. The number of subsampled reads (i.e. 20,000) for the downstream quality filtering pipeline is indicated by a solid black line. See Supplemental Table S1 for the total “number of 18S reads” per sample. [file peerj-07-6379-s002.pdf]
