# Supplementary material for: Digging for DNA at depth: rapid universal metabarcoding surveys (RUMS) as a tool to detect coral reef biodiversity across a depth gradient
Source: PeerJ. 2019 Feb 6;7:e6379. doi: 10.7717/peerj.6379 (PMC6368839; doi:10.7717/peerj.6379)
Supplement: Supplemental Information 3 — 18S rRNA raw reads immediately after sequencing by replicate for sediment samples taken at two coral reefs in Okinawa, Japan (depth: 10 m, 20 m, 30 m, 40 m). “Assigned unique reads” refers to the number of unique reads assigned at the family-level in MEGAN vers. 5.11.3 (MEGAN run parameters outlined in the Materials and methods); “total unique reads” refers to the number of dereplicated sequences after quality filtering (QF). [file peerj-07-6379-s003.docx]

| Sample_ID | Location | Year | Latitude | Longitude | Depth | Number of 18S reads | Post-QF 18S *N* > 2  assigned unique reads (total unique reads) |
| --- | --- | --- | --- | --- | --- | --- | --- |
| AWFS_F16_0427 | Cape Hedo | 2016 | 26.87228 | 128.26652 | 10 m | 54,725 | 597 (805) |
| AWFS_F16_0428 | Cape Hedo | 2016 | 26.87228 | 128.26652 | 10 m | 101,967 | 564 (677) |
| 39 | Cape Hedo | 2016 | 26.87228 | 128.26652 | 10 m | 98,974 | 462 (913) |
| AWFS_F16_0429 | Cape Hedo | 2016 | 26.87228 | 128.26652 | 20 m | 2,467 | 0 (0) |
| AWFS_F16_0430 | Cape Hedo | 2016 | 26.87228 | 128.26652 | 20 m | 65,430 | 609 (704) |
| 42 | Cape Hedo | 2016 | 26.87228 | 128.26652 | 20 m | 72,601 | 235 (1,047) |
| AWFS_F16_0431 | Cape Hedo | 2016 | 26.87228 | 128.26652 | 30 m | 36,400 | 270 (884) |
| AWFS_F16_0432 | Cape Hedo | 2016 | 26.87228 | 128.26652 | 30 m | 44,574 | 643 (681) |
| 45 | Cape Hedo | 2016 | 26.87228 | 128.26652 | 30 m | 66,602 | 408 (1,322) |
| 46 | Cape Hedo | 2016 | 26.87228 | 128.26652 | 30 m | 123,482 | 472 (949) |
| AWFS_F16_0433 | Cape Hedo | 2016 | 26.87228 | 128.26652 | 40 m | 83,517 | 685 (697) |
| AWFS_F16_0434 | Cape Hedo | 2016 | 26.87228 | 128.26652 | 40 m | 28,143 | 403 (1,099) |
| 49 | Cape Hedo | 2016 | 26.87228 | 128.26652 | 40 m | 46,653 | 404 (1,279) |
| 50 | Cape Hedo | 2016 | 26.87228 | 128.26652 | 40 m | 98,122 | 298 (1,083) |
| SED120 | Cape Hedo | 2017 | 26.87228 | 128.26652 | 10 m | 90,789 | 253 (989) |
| SED121 | Cape Hedo | 2017 | 26.87228 | 128.26652 | 10 m | 202,696 | 602 (669) |
| SED122 | Cape Hedo | 2017 | 26.87228 | 128.26652 | 10 m | 104,785 | 563 (788) |
| SED123 | Cape Hedo | 2017 | 26.87228 | 128.26652 | 10 m | 409,178 | 668 (697) |
| SED124 | Cape Hedo | 2017 | 26.87228 | 128.26652 | 20 m | 432,792 | 338 (757) |
| SED125 | Cape Hedo | 2017 | 26.87228 | 128.26652 | 20 m | 86,904 | 611 (923) |
| SED126 | Cape Hedo | 2017 | 26.87228 | 128.26652 | 20 m | 8,366 | 0 (0) |
| SED127 | Cape Hedo | 2017 | 26.87228 | 128.26652 | 20 m | 106,612 | 561 (961) |
| SED128 | Cape Hedo | 2017 | 26.87228 | 128.26652 | 30 m | 134,797 | 61 (855) |
| SED129 | Cape Hedo | 2017 | 26.87228 | 128.26652 | 30 m | 123,997 | 117 (887) |
| SED130 | Cape Hedo | 2017 | 26.87228 | 128.26652 | 30 m | 154,677 | 507 (905) |
| SED131 | Cape Hedo | 2017 | 26.87228 | 128.26652 | 30 m | 24,997 | 1,018 (1,039) |
| SED132 | Cape Hedo | 2017 | 26.87228 | 128.26652 | 40 m | 103,213 | 402 (998) |
| SED133 | Cape Hedo | 2017 | 26.87228 | 128.26652 | 40 m | 75,589 | 576 (846) |
| SED134 | Cape Hedo | 2017 | 26.87228 | 128.26652 | 40 m | 66,053 | 425 (791) |
| SED135 | Cape Hedo | 2017 | 26.87228 | 128.26652 | 40 m | 77,112 | 97 (738) |
| AWFS_F16_0437 | Rukan | 2016 | 26.09961 | 127.53962 | 10 m | 28,112 | 626 (800) |
| AWFS_F16_0438 | Rukan | 2016 | 26.09961 | 127.53962 | 10 m | 93,017 | 94 (613) |
| 57 | Rukan | 2016 | 26.09961 | 127.53962 | 10 m | 73,859 | 299 (1,123) |
| 58 | Rukan | 2016 | 26.09961 | 127.53962 | 10 m | 89,028 | 334 (1,058) |
| AWFS_F16_0439 | Rukan | 2016 | 26.09961 | 127.53962 | 20 m | 63,542 | 466 (1,045) |
| AWFS_F16_0440 | Rukan | 2016 | 26.09961 | 127.53962 | 20 m | 29,492 | 518 (982) |
| 61 | Rukan | 2016 | 26.09961 | 127.53962 | 20 m | 52,938 | 748 (1,356) |
| 62 | Rukan | 2016 | 26.09961 | 127.53962 | 20 m | 25,169 | 645 (683) |
| AWFS_F16_0441 | Rukan | 2016 | 26.09961 | 127.53962 | 30 m | 49,407 | 490 (1,024) |
| AWFS_F16_0442 | Rukan | 2016 | 26.09961 | 127.53962 | 30 m | 56,887 | 596 (780) |
| 65 | Rukan | 2016 | 26.09961 | 127.53962 | 30 m | 59,038 | 162 (998) |
| 66 | Rukan | 2016 | 26.09961 | 127.53962 | 30 m | 40,585 | 665 (1,444) |
