# Supplementary material for: Digging for DNA at depth: rapid universal metabarcoding surveys (RUMS) as a tool to detect coral reef biodiversity across a depth gradient
Source: PeerJ. 2019 Feb 6;7:e6379. doi: 10.7717/peerj.6379 (PMC6368839; doi:10.7717/peerj.6379)
Supplement: Supplemental Information 4 — List of all combined families in alphabetical order assigned in MEGAN vers. 5.11.3, including order and class hierarchy, based on 18S rRNA sequences from sediment sampled at two coral reefs (10 m, 20 m, 30 m, 40 m) in Okinawa, Japan. [file peerj-07-6379-s004.docx]

| Class | Order | Family |
| --- | --- | --- |
| Acoela |  | Haploposthiidae |
| Acoela |  | Isodiametridae |
| Agaricomycetes | Boletales | Boletaceae |
| Anthozoa | Scleractinia | Pocilloporidae |
| Anthozoa | Zoantharia | Parazoanthidae |
| Appendicularia | Copelata | Oikopleuridae |
| Arachnida | Trombidiformes | Halacaridae |
| Ascidiacea | Aplousobranchia | Didemnidae |
| Ascidiacea | Phlebobranchia | Ascidiidae |
| Ascidiacea | Phlebobranchia | Perophoridae |
| Ascidiacea | Stolidobranchia | Pyuridae |
| Ascidiacea | Stolidobranchia | Styelidae |
| Bacillariophyceae | Achnanthales | Cocconeidaceae |
| Bacillariophyceae | Bacillariales | Bacillariaceae |
| Bacillariophyceae | Biddulphiales | Biddulphiaceae |
| Bacillariophyceae | Chaetocerotanae incertae sedis | Attheyaceae |
| Bacillariophyceae | Chaetocerotanae incertae sedis | Chaetocerotaceae |
| Bacillariophyceae | Cymatosirales | Cymatosiraceae |
| Bacillariophyceae | Eunotiales | Eunotiaceae |
| Bacillariophyceae | Fragilariales | Fragilariaceae |
| Bacillariophyceae | Licmophorales | Licmophoraceae |
| Bacillariophyceae | Naviculales | Diploneidaceae |
| Bacillariophyceae | Naviculales | Naviculaceae |
| Bacillariophyceae | Naviculales | Pleurosigmataceae |
| Bacillariophyceae | Naviculales | Sellaphoraceae |
| Bacillariophyceae | Naviculales | Amphipleuraceae |
| Bacillariophyceae | Rhopalodiales | Rhopalodiaceae |
| Bacillariophyceae | Surirellales | Entomoneidaceae |
| Bacillariophyceae | Thalassiophysales | Catenulaceae |
| Bacillariophyceae | Thalassiosirales | Thalassiosiraceae |
| Bacillariophyceae | Triceratiales | Plagiogrammaceae |
| Bacillariophyceae | Achnanthales | Achnanthidiaceae |
| Bicoecea | Bicoecida | Bicosoecidae |
| Bivalvia | Arcida | Arcidae |
| Bivalvia | Chamidae | Chamidae |
| Bivalvia | Lucinida | Lucinidae |
| Bivalvia | Mytilida | Mytilidae |
| Bivalvia | Venerida | Veneridae |
| Bivalvia |  | Gastrochaenidae |
| Bivalvia |  | Lasaeidae |
| Calcarea | Clathrinida | Clathrinidae |
| Calcarea | Leucosolenida | Grantiidae |
| Calcarea | Leucosolenida | Sycettidae |
| Catenulida |  | Catenulidae |
| Centrohelea | Centrohelida | Acanthocystidae |
| Chlorodendrophyceae | Chlorodendrales | Chlorodendraceae |
| Choanoflagellatea | Acanthoecida | Acanthoecidae |
| Chromadorea | Chromadorida | Chromadoridae |
| Chromadorea | Chromadorida | Cyatholaimidae |
| Chromadorea | Desmodorida | Draconematidae |
| Chromadorea | Desmodorida | Desmodoridae |
| Chromadorea | Desmoscolecida | Desmoscolecidae |
| Chromadorea | Plectida | Leptolaimidae |
| Chromadorea | Desmodorida | Microlaimidae |
| Chrysophyceae | Ochromonadales | Chromulinaceae |
| Chrysophyceae | Synurales | Mallomonadaceae |
| Chrysophyceae | Ochromonadales | Ochromonadaceae |
| Chrysophyceae | Ochromonadales | Paraphysomonadaceae |
| Chytridiomycetes | Chytridiales | Chytriomycetaceae |
| Chytridiomycetes | Lobulomycetales | Lobulomycetaceae |
| Compsopogonophyceae | Erythropeltidales | Erythrotrichiaceae |
| Compsopogonophyceae | Rhodochaetales | Rhodochaetaceae |
| Conoidasida | Eugregarinorida | Gregarinidae |
| Conoidasida | Eugregarinorida | Selenidiidae |
| Conoidasida | Eugregarinorida | Lecudinidae |
| Cryptophyceae | Pyrenomonadales | Pyrenomonadaceae |
| Demospongiae | Axinellida | Axinellidae |
| Demospongiae | Axinellida | Raspailiidae |
| Demospongiae | Chondrillida | Halisarcidae |
| Demospongiae | Clionaida | Spirastrellidae |
| Demospongiae | Desmacellida | Desmacellidae |
| Demospongiae | Haplosclerida | Petrosiidae |
| Demospongiae | Tetractinellida | Theonellidae |
| Demospongiae | Poecilosclerida | Microcionidae |
| Dictyochophyceae | Pinguiochrysidales | Pinguiochrysidaceae |
| Dinoflagellata | Macrodasyida | Thaumastodermatidae |
| Dinophyceae | Dinophyceae incertae sedis | Amphidomataceae |
| Dinophyceae | Gonyaulacales | Ceratocoryaceae |
| Dinophyceae | Gonyaulacales | Gonyaulacaceae |
| Dinophyceae | Gymnodiniales | Gymnodiniaceae |
| Dinophyceae | Gymnodiniales | Kareniaceae |
| Dinophyceae | Peridiniales | Heterocapsaceae |
| Dinophyceae | Peridiniales | Peridiniaceae |
| Dinophyceae | Prorocentrales | Prorocentraceae |
| Dinophyceae | Suessiales | Symbiodiniaceae |
| Dinophyceae | Syndiniales | Amoebophryaceae |
| Discosea | Dactylopodida | Paramoebidae |
| Dothideomycetes | Pleosporales | Didymellaceae |
| Dothideomycetes | Pleosporales | Didymosphaeriaceae |
| Enoplea | Enoplida | Ironidae |
| Enoplea | Enoplida | Oncholaimidae |
| Enoplea | Enoplida | Oxystominidae |
| Enteropneusta |  | Harrimaniidae |
| Florideophyceae | Corallinales | Corallinaceae |
| Florideophyceae | Corallinales | Hapalidiaceae |
| Florideophyceae | Gelidiales | Gelidiaceae |
| Florideophyceae | Gigartinales | Cystocloniaceae |
| Florideophyceae | Nemastomatales | Nemastomataceae |
| Florideophyceae | Peyssonneliales | Peyssonneliaceae |
| Florideophyceae | Rhodymeniales | Champiaceae |
| Gastropoda | Littorinimorpha | Caecidae |
| Gastropoda | Littorinimorpha | Elachisinidae |
| Gastropoda |  | Parhedylidae |
| Glissodiscea | Planomonadida | Planomonadidae |
| Goniomonadophyceae | Goniomonadales | Goniomonadaceae |
| Gromiidea | Gromiida | Gromiidae |
| Gymnolaemata | Cheilostomatida | Schizoporellidae |
| Gymnolaemata | Cheilostomatida | Romancheinidae |
| Gymnolaemata | Cheilostomatida | Membraniporidae |
| Gymnolaemata | Cheilostomatida | Flustridae |
| Heterotrichea | Heterotrichida | Blepharismidae |
| Heterotrichea | Heterotrichida | Condylostomatidae |
| Heterotrichea | Heterotrichida | Peritromidae |
| Heterotrichea | Heterotrichida | Folliculinidae |
| Hexanauplia | Calanoida | Aetideidae |
| Hexanauplia | Calanoida | Pseudocyclopidae |
| Hexanauplia | Cyclopoida | Cyclopidae |
| Hexanauplia | Cyclopoida | Pseudanthessiidae |
| Hexanauplia | Harpacticoida | Ameiridae |
| Hexanauplia | Harpacticoida | Canthocamptidae |
| Hexanauplia | Harpacticoida | Ectinosomatidae |
| Hexanauplia | Harpacticoida | Miraciidae |
| Hexanauplia | Siphonostomatoida | Asterocheridae |
| Hexanauplia | Harpacticoida | Dactylopusiidae |
| Hydrozoa | Leptothecata | Campanulariidae |
| Hydrozoa | Leptothecata | Tiaropsidae |
| Hyphochytriomycetes | Hyphochytriales | Rhizidiomycetaceae |
| Imbricatea | Thaumatomonadida | Thaumatomonadidae |
| Karyorelictea | Protostomatida | Trachelocercidae |
| Labyrinthulea | Labyrinthulida | Labyrinthula |
| Labyrinthulea | Thraustochytriales | Thraustochytriaceae |
| Leotiomycetes | Helotiales | Sclerotiniaceae |
| Litostomatea | Haptorida | Lacrymariidae |
| Litostomatea | Haptorida | Tracheliidae |
| Litostomatea | Pleurostomatida | Litonotidae |
| Magnoliopsida | Alismatales | Zosteraceae |
| Magnoliopsida | Brassicales | Brassicaceae |
| Magnoliopsida | Lamiales | Acanthaceae |
| Mamiellophyceae | Mamiellales | Mamiellaceae |
| Oligohymenophorea | Philasterida | Loxocephalidae |
| Oligohymenophorea | Philasterida | Philasteridae |
| Oligotrichea | Choreotrichida | Strombidinopsidae |
| Oligotrichea | Halteriida | Halteriidae |
| Oligotrichea | Oligotrichida | Strombidiidae |
| Ostracoda | Podocopida | Cytheruridae |
| Ostracoda | Podocopida | Eucytheridae |
| Ostracoda | Podocopida | Loxoconchidae |
| Ostracoda | Podocopida | Thaerocytheridae |
| Ostracoda | Podocopida | Xestoleberididae |
| Ostracoda | Podocopida | Cushmanideidae |
| Palaeonemertea |  | Cephalothrichidae |
| Pavlovophyceae | Pavlovales | Pavlovaceae |
| Pedinophyceae | Pedinomonadales | Pedinomonadaceae |
| Peronosporea | Saprolegniales | Haliphthoraceae |
| Phaeophyceae | Dictyotales | Dictyotaceae |
| Phascolosomatidea | Phascolosomatida | Phascolosomatidae |
| Phytomyxea | Plasmodiophorida | Plasmodiophoridae |
| Polychaeta | Capitellida | Capitellidae |
| Polychaeta | Capitellida | Maldanidae |
| Polychaeta | Eunicida | Dorvilleidae |
| Polychaeta | Eunicida | Eunicidae |
| Polychaeta | Phyllodocida | Chrysopetalidae |
| Polychaeta | Phyllodocida | Glyceridae |
| Polychaeta | Phyllodocida | Hesionidae |
| Polychaeta | Phyllodocida | Pholoidae |
| Polychaeta | Phyllodocida | Phyllodocidae |
| Polychaeta | Phyllodocida | Polynoidae |
| Polychaeta | Phyllodocida | Sigalionidae |
| Polychaeta | Phyllodocida | Syllidae |
| Polychaeta | Sabellida | Oweniidae |
| Polychaeta | Sabellida | Sabellidae |
| Polychaeta | Sabellida | Serpulidae |
| Polychaeta | Spionida | Chaetopteridae |
| Polychaeta | Spionida | Poecilochaetidae |
| Polychaeta | Spionida | Spionidae |
| Polychaeta | Terebellida | Terebellidae |
| Polychaeta |  | Nerillidae |
| Polychaeta |  | Opheliidae |
| Polychaeta |  | Paraonidae |
| Polychaeta |  | Protodrilidae |
| Polyplacophora | Chitonida | Cryptoplacidae |
| Porphyridiophyceae | Porphyridiales | Porphyridiaceae |
| Prostomatea | Prorodontida | Placidae |
| Prostomatea | Prorodontida | Colepidae |
| Prymnesiophyceae | Coccolithales | Coccolithaceae |
| Prymnesiophyceae | Coccosphaerales | Calyptrosphaeraceae |
| Prymnesiophyceae | Prymnesiales | Prymnesiaceae |
| Pyramimonadophyceae | Pseudoscourfieldiales | Pycnococcaceae |
| Raphidophyceae | Chattonellales | Chattonellaceae |
| Rhabditophora | Dolichomicrostomida | Microstomidae |
| Rhabditophora | Lecithoepitheliata | Gnosonesimidae |
| Rhabditophora | Proseriata | Monocelididae |
| Rhabditophora | Proseriata | Otoplanidae |
| Rhabditophora | Rhabdocoela | Polycystididae |
| Rhabditophora | Rhabdocoela | Promesostomidae |
| Saccharomycetes | Saccharomycetales | Metschnikowiaceae |
| Sarcomonadea | Cercomonadida | Cercomonadidae |
| Sordariomycetes | Sordariomycetes incertae sedis | Koralionastetaceae |
| Spirotrichea | Euplotida | Certesiidae |
| Spirotrichea | Euplotida | Euplotidae |
| Spirotrichea | Euplotida | Discocephalidae |
| Spirotrichea | Urostylida | Pseudourostylidae |
| Spirotrichea |  | Holostichidae |
| Spirotrichea |  | Oxytrichidae |
| Spirotrichea |  | Pseudokeronopsidae |
| Stenolaemata | Cyclostomatida | Densiporidae |
| Stylonematophyceae | Stylonematales | Stylonemataceae |
| Thaliacea | Doliolida | Doliolidae |
| Thecomonadea | Apusomonadida | Apusomonadidae |
| Trebouxiophyceae | Chlorellales | Chlorellaceae |
| Tubulinea | Arcellinida | Centropyxidae |
| Ulvophyceae | Bryopsidales | Bryopsidaceae |
| Ulvophyceae | Cladophorales | Cladophoraceae |
| Ulvophyceae | Ulotrichales | Ulotrichaceae |
| Ulvophyceae | Ulvales | Cloniophoraceae |
| Ulvophyceae | Ulvales | Phaeophilaceae |
| Ulvophyceae | Ulvales | Ulvellaceae |
|  | Chaetonotida | Chaetonotidae |
|  | Colpodellida | Colpodellidae |
|  | Duboscquellales | Duboscquellaceae |
|  | Macrodasyida | Dactylopodolidae |
|  | Macrodasyida | Lepidodasyidae |
